# Genetic parameters of feather corticosterone and fault bars and correlations with production traits in turkeys (*Meleagris gallopavo*)

**DOI:** 10.1038/s41598-022-26734-6

**Published:** 2023-01-02

**Authors:** Emily M. Leishman, Ryley J. Vanderhout, Emhimad A. Abdalla, Nienke van Staaveren, Anna Naim, Shai Barbut, Benjamin J. Wood, Alexandra Harlander-Matauschek, Christine F. Baes

**Affiliations:** 1 Department of Animal Biosciences, Centre for the Genetic Improvement of Livestock, University of Guelph, Guelph, ON N1G 2W1 Canada; 2 Department of Animal Biosciences, Campbell Centre for the Study of Animal Welfare, University of Guelph, Guelph, ON N1G 2W1 Canada; 3 Department of Food Science, University of Guelph, Guelph, ON N1G 2W1 Canada; 4 Hybrid Turkeys, Suite C, 650 Riverbend Drive, Kitchener, ON N2K 3S2 Canada; 5 School of Veterinary Science, University of Queensland, Gatton, QLD 4343 Australia; 6 Institute of Genetics, Vetsuisse Faculty, University of Bern, 3001 Bern, Switzerland

**Keywords:** Agricultural genetics, Animal breeding, Animal physiology, Neuroendocrinology

## Abstract

Robustness can refer to an animal’s ability to overcome perturbations. Intense selection for production traits in livestock has resulted in reduced robustness which has negative implications for livability as well as production. There is increasing emphasis on improving robustness through poultry breeding, which may involve identifying novel phenotypes that could be used in selection strategies. The hypothalamic–pituitary–adrenal (HPA) axis and associated hormones (e.g., corticosterone) participate in many metabolic processes that are related to robustness. Corticosterone can be measured non-invasively in feathers (FCORT) and reflects the average HPA axis activity over the feather growing period, however measurement is expensive and time consuming. Fault bars are visible feather deformities that may be related to HPA axis activity and may be a more feasible indicator trait. In this study, we estimated variance components for FCORT and fault bars in a population of purebred turkeys as well as their genetic and partial phenotypic correlations with other economically relevant traits including growth and efficiency, carcass yield, and meat quality. The estimated heritability for FCORT was 0.21 ± 0.07 and for the fault bar traits (presence, incidence, severity, and index) estimates ranged from 0.09 to 0.24. The genetic correlation of FCORT with breast weight, breast meat yield, fillet weight, and ultimate pH were estimated at −0.34 ± 0.21, −0.45 ± 0.23, −0.33 ± 0.24, and 0.32 ± 0.24, respectively. The phenotypic correlations of FCORT with breast weight, breast meat yield, fillet weight, drum weight, and walking ability were −0.16, −0.23, −0.18, 0.17, and 0.21, respectively. Some fault bar traits showed similar genetic correlations with breast weight, breast meat yield, and walking ability but the magnitude was lower than those with FCORT. While the dataset is limited and results should be interpreted with caution, this study indicates that selection for traits related to HPA axis activity is possible in domestic turkeys. Further research should focus on investigating the association of these traits with other robustness-related traits and how to potentially implement these traits in turkey breeding.

## Introduction

In livestock production, robustness can be defined as an animal’s ability to maintain production while being resilient to perturbations allowing for high production in many different environments^1^. Robustness is a critical issue in poultry breeding because of its implications for profitability, health, and wellbeing^2^. Intense selection for production traits now has a well-established relationship with reduced robustness^3^. This trade-off between robustness and production is described in the “resource allocation theory” which states that the resources of an individual are limited, and allocation of these resources should optimize the individual’s fitness in its environment^4^. Breeding for increased production (e.g., breast yield) is believed to compromise robustness and increase behavioural, physiological, and immunological disorders^2^. Traditionally, changes in housing and management are used to try and reduce these problems^2^, but strategies to improve poultry robustness through breeding have also been suggested. The most common strategies include reaction norm analyses^5^ or direct selection for robustness-related traits like disease resistance^6^ or leg health^7^.

Another strategy to incorporate robustness into animal breeding is to investigate the genetics of the hypothalamic–pituitary–adrenal (HPA) axis^8,9^. The HPA axis is connected to robustness because of its pivotal role in regulating energy balance (influencing e.g., behaviour, appetite, adaptation, inflammatory response) and the neuroendocrine stress response^8–11^. In poultry, higher HPA axis activity has been demonstrated in genetic lines that are more resistant to disease/parasites^12^ and more resilient to environmental challenges^13^.Domestication and intense selection for production traits in livestock tends to result in reduced HPA axis activity, meaning lower levels of glucocorticoids are produced. Over the past few years, there has been a shift in how we interpret glucocorticoid levels in animals; specifically, challenging the assumption that low glucocorticoid levels indicate an animal that is healthy or doing well^14^. Low HPA axis activity has been associated with reduced disease and parasite resistance^12,15,16^, reduced early survival^17^, poorer adaptation to environmental challenges^13,18^, and poorer confirmation/leg soundness^19^. Therefore, it may be possible to improve robustness by selecting for increased HPA axis activity since individuals with higher HPA axis activity may be more resilient to the aforementioned challenges^8^.

HPA axis activity is commonly quantified by measuring end-products such as glucocorticoid hormones^20^. In birds, the predominant glucocorticoid hormone is corticosterone (CORT)^20^ which can be measured in plasma offering an acute perspective on the circulating level of corticosterone. A more longitudinal and retrospective measure of HPA axis activity is feather corticosterone (FCORT)^21^ which benefits from minimally-invasive collection and represents a long period of time within a sample^22,23^. Another accessible and cost-effective phenotype might be the use of feather fault bars. Fault bars are visible feather deformities that run perpendicular to the rachis and are thought to be related to perturbations such as predators or nutritional deficiencies^24,25^. Although the development of these fault bars is typically attributed to acute perturbations, the incidence of fault bars has been shown to be positively correlated with FCORT^26,27^. There may be a genetic component that dictates the propensity for certain individuals to develop fault bars^28^.

Previously, we showed that FCORT levels differ between different turkey genetic lines which may indicate underlying genetic differences^29,30^. Heritability of FCORT or fault bars has yet to be estimated; however, several estimates exist for plasma corticosterone in various domestic and wild bird species. Heritability of plasma corticosterone were estimated for bird species including turkeys^31^, laying hens^32^, quails^33^, zebra finches^34^, and owls^35^ (range: 0.05–0.30), suggesting that selection for this trait is possible. However, it should be noted that only one of these studies referred to turkeys and this study was conducted almost 50 years ago. The continued genetic selection over the past decade may have changed heritability of corticosterone, and to date, there are a lack of heritability estimates for fault bars in any avian species.

While these novel phenotypes related to HPA axis activity may be a possibility for improving robustness, correlations with economically important traits must be considered^36,37^. Due to the widespread influence of the HPA axis on many physiological processes, it is conceivable that FCORT and/or fault bars may have favourable or unfavourable relationships with traits such as yield, efficiency, and meat quality^38,39^. While unfavourable relationships between HPA axis activity and production have been well described in other species, there is less known about this relationship in domestic turkeys.

The objective of the present study was to estimate heritability of FCORT and fault bars using data collected from 1131 male turkeys from three purebred genetic lines. Additionally, we described the genetic and phenotypic correlations of FCORT and fault bars with economically important traits in turkey production related to growth and efficiency, carcass yield, and meat quality. This study was conducted as part of a larger project to develop novel welfare phenotypes in domestic turkey breeding^40^.

## Results

Feathers were collected from 1131 turkeys from three different genetic lines (A, B, and C). Due to the observational design of the study, not all birds have records for all growth and efficiency, carcass, and meat quality traits, and the number varies depending on the genetic line. A complete breakdown of the distribution of observations among the genetic lines can be found in Supplementary Table S1.

### Description of feather traits

Across all lines, FCORT ranged from 0.14 to 4.16 pg/mg (N = 1131). The average ± standard deviation (SD) for FCORT across all individuals was 1.24 ± 0.55 pg/mg. The percentage of birds exhibiting fault bars (fault bar presence) was approximately 48% (N = 523) and the remaining 52% of birds did not exhibit any fault bars (N = 562). In birds that exhibited fault bars, the incidence of fault bars (FB incidence) ranged from 1 to 12 (mean ± SD = 1.9 ± 1.3). For FB severity, the majority of birds affected with fault bars received a score 2 (light bar, 51%, N = 267), followed by score 4 (severe bar, 31%, N = 163), and lastly score 3 (moderate bar, 18%, N = 93).

### Heritabilities

Heritability estimates for the feather traits are presented in Table 1. Heritability estimates for the studied growth and efficiency, carcass, and meat quality traits can be found in Vanderhout et al.^41^. The reported heritability estimates in this study are from the univariate models for each trait.

**Table 1:** Heritability (diagonal), genetic (above diagonal), and partial phenotypic (below diagonal)^1^ correlations among the feather traits: feather corticosterone (FCORT) and fault bar (FB) traits (N = 1078).

	FCORT	FB presence	FB incidence	FB severity	FB index
FCORT	0.21 ± 0.07	0.39 ± 0.74	−0.07 ± 0.34	0.14 ± 0.66	−0.01 ± 0.60
FB presence	−0.11	0.09 ± 0.06	0.86 ± 0.63	1.00 ± 0.38	1.00 ± 0.19
FB incidence	−0.10	0.60^b^	0.24 ± 0.09	0.68 ± 0.43	0.98 ± 0.17
FB severity	−0.14	0.83^b^	0.48^b^	0.11 ± 0.07	0.87 ± 0.29
FB index	−0.16^a^	0.67^b^	0.87^b^	0.76^b^	0.21 ± 0.08

^1^Significant partial phenotypic correlations are denoted by letter superscripts where a = *p* < 0.05 and b = *p* < 0.0001.

### Correlations

Among the feather traits, FCORT had a significant partial phenotypic correlation with fault bar index (−0.16, Table 1), but low and insignificant partial phenotypic correlations with the remaining fault bar traits (r_p_ = −0.11 to −0.14). Genetic correlations between FCORT and the fault bar traits varied from -0.07 to 0.39 with large standard errors. The other fault bar traits showed moderate to high partial phenotypic and genetic correlations among them. The phenotypic correlations ranged from 0.48 to 0.87 and the genetic correlations ranged from 0.68 to 1.00 (SE range: 0.17–0.63).

Genetic and partial phenotypic correlations of feather traits and growth and efficiency traits are presented in Table 2. In general, both FCORT and fault bar traits had low and insignificant phenotypic correlations with body weight, walking score and feed conversion ratio, except for walking score with FCORT (0.21) and fault bar incidence (0.17). Genetic correlations among FCORT and fault bar traits with body weight, walking score and feed conversion ratio ranged from zero to 0.83, but the SE were generally high.

**Table 2:** Genetic and partial phenotypic correlations of feather corticosterone (FCORT) and fault bar (FB) traits with growth and efficiency traits in turkeys (N = 408–1130 depending on trait). Growth and efficiency traits include body weight at 12 weeks of age (BW12, kg), body weight just prior to slaughter (BW20, kg), walking score (WS20), and feed conversion ratio (FCR).

Trait	BW12	BW20	WS20	FCR
N	1130	1114	1,126	408
**Partial phenotypic correlation**^**1**^				
FCORT	0.02	−0.01	0.21^a^	−0.05
FB presence	0.05	−0.07	0.07	0.02
FB incidence	−0.02	−0.08	0.17^a^	−0.02
FB severity	0.11	−0.01	0.07	0.03
FB index	0.04	−0.04	0.13	0.00
**Additive genetic correlation**				
FCORT	−0.03 ± 0.17	0.00 ± 0.18	0.11 ± 0.25	0.29 ± 0.25
FB presence	0.12 ± 0.33	0.10 ± 0.52	0.83 ± 1.00	0.71 ± 1.00
FB incidence	0.08 ± 0.17	−0.06 ± 0.17	0.23 ± 0.25	0.03 ± 0.24
FB severity	−0.01 ± 0.27	−0.01 ± 0.27	0.59 ± 0.52	0.36 ± 0.53
FB index	0.03 ± 0.18	−0.09 ± 0.19	0.38 ± 0.28	0.10 ± 0.36

^1^Significant partial phenotypic correlations are denoted by letter superscripts where a = *p* < 0.05 and b = *p* < 0.0001.

When considering specific carcass traits, FCORT showed low-moderate partial phenotypic correlations with the different cut weights and yield (Table 3). There were significant partial phenotypic correlations with total breast weight (−0.16), breast meat yield (−0.23), fillet weight (−0.18), and drum weight (0.17). None of the other fault bar traits showed significant partial phenotypic correlations with the carcass traits. Genetic correlation estimates between FCORT and fault bar traits with carcass traits fluctuated from low to high; however, SE were also high for most estimates. Nevertheless, FCORT showed a moderate genetic correlation with total breast weight (−0.34 ± 0.21), breast meat yield (−0.45 ± 0.23), and fillet weight (−0.33 ± 0.24). Fault bar incidence and fault bar index were genetically correlated with total breast weight (fault bar incidence: −0.23 ± 0.18; fault bar index: (−0.20 ± 0.18), and breast meat yield (fault bar incidence only: −0.26 ± 0.21). FCORT and the fault bar traits demonstrated low partial phenotypic correlations with the meat quality traits (Table 4), and, apart from some correlations with drip loss and cooking loss, none of these correlations were significant. Genetic correlation estimates between FCORT and the meat quality traits were low to moderate, suggesting a moderate correlation between FCORT and ultimate pH (0.32 ± 0.24) as well as fault bar index and a* (−0.31 ± 0.24). However, it should be acknowledged that the standard errors of all estimates were high.

**Table 3:** Genetic and partial phenotypic correlations of feather corticosterone (FCORT) and fault bar (FB) traits with carcass traits in turkeys (N = 771–1060 depending on trait). Carcass traits include Breast_Total_ (fillet + tender, kg), breast meat yield (BMY, %), fillet weight (kg), tender weight (kg), thigh weight (kg), and drum weight (kg).

Trait	Breast_Total_	BMY	Fillets	Tenders	Thighs	Drums
N	1060	1045	773	771	832	811
**Partial phenotypic correlation**^**1**^						
FCORT	−0.16^a^	−0.23^a^	−0.18^a^	0.00	0.04	0.17^a^
FB presence	−0.09	−0.07	−0.09	−0.02	−0.07	−0.13
FB incidence	−0.13	−0.13	−0.13	−0.06	−0.08	−0.08
FB severity	0.00	0.01	−0.02	0.09	−0.04	−0.09
FB index	−0.08	−0.09	−0.10	0.03	−0.07	−0.07
**Additive genetic correlation**						
FCORT	−0.34 ± 0.21	−0.45 ± 0.23	−0.33 ± 0.24	0.01 ± 0.21	0.05 ± 0.24	0.11 ± 0.38
FB presence	−0.07 ± 1.00	−0.15 ± 0.39	−0.23 ± 0.51	0.65 ± 0.97	0.04 ± 0.42	0.14 ± 0.57
FB incidence	−0.23 ± 0.18	−0.26 ± 0.21	−0.11 ± 0.23	−0.12 ± 0.20	0.11 ± 0.22	0.05 ± 0.20
FB severity	−0.02 ± 0.27	−0.02 ± 0.32	−0.07 ± 0.37	0.55 ± 0.70	0.18 ± 0.47	0.13 ± 0.34
FB index	−0.20 ± 0.18	−0.20 ± 0.27	−0.10 ± 0.23	0.02 ± 0.25	0.17 ± 0.39	0.09 ± 0.23

^1^Significant partial phenotypic correlations are denoted by letter superscripts where a = *p* < 0.05 and b = *p* < 0.0001.

**Table 4:** Genetic and partial phenotypic correlations of feather corticosterone (FCORT) and fault bar (FB) traits with meat quality traits in turkeys (N = 734–1068 depending on trait). Meat quality traits include lightness (L*), redness (a*), yellowness (b*), pH_ultimate_, drip loss (DL, %), cooking loss (CL, %), and shear force (SF, N).

Trait	L*	a*	b*	pH_ultimate_	DL	CL	SF
N	1068	1068	1068	834	837	834	734
**Partial phenotypic correlation**^**1**^							
FCORT	−0.07	−0.02	−0.02	0.06	−0.17^a^	−0.11	0.08
FB presence	−0.03	0.00	−0.02	−0.08	−0.04	0.15^a^	0.09
FB incidence	−0.05	−0.05	−0.13	0.02	0.01	0.02	0.08
FB severity	−0.04	0.04	−0.01	−0.11	−0.01	0.16^a^	0.07
FB index	−0.05	−0.03	−0.10	−0.04	0.03	0.11	0.07
**Additive genetic correlation**							
FCORT	−0.18 ± 0.20	−0.07 ± 0.22	0.19 ± 0.21	0.32 ± 0.24	−0.07 ± 0.80	−0.70 ± 1.00	0.14 ± 0.60
FB presence	0.05 ± 0.48	−0.40 ± 0.48	−0.06 ± 0.50	0.51 ± 0.63	−0.15 ± 1.00	−0.99 ± 0.13	−0.21 ± 1.00
FB incidence	0.00 ± 0.20	−0.30 ± 0.22	−0.15 ± 0.20	0.32 ± 0.38	−0.76 ± 1.00	−0.77 ± 1.00	−0.30 ± 0.46
FB severity	0.19 ± 0.42	−0.20 ± 0.33	−0.03 ± 0.34	0.35 ± 0.54	0.32 ± 1.00	−0.63 ± 1.00	−0.32 ± 0.74
FB index	0.15 ± 0.21	−0.31 ± 0.24	−0.08 ± 0.22	0.29 ± 0.42	−0.16 ± 1.00	−0.58 ± 0.96	0.29 ± 0.42

^1^Significant partial phenotypic correlations are denoted by letter superscripts where a = *p* < 0.05 and b = *p* < 0.0001.

## Discussion

In the present study, we estimated variance components for novel feather traits (FCORT and fault bars) associated with stress and energy balance in domestic turkeys. The genetic and phenotypic relationships among these novel traits, production traits, carcass traits, and meat quality traits were evaluated to inform their potential for inclusion in breeding program.

Although there are no published studies of heritability estimates for FCORT, the results for this trait were similar to estimates from plasma corticosterone traits measured in laying hens (h^2^ = 0.05)^32^, quails (h^2^ = 0.05–0.30)^33^, zebra finches (h^2^ = 0.04–0.27)^34^ after a restraint stressor was applied. More specific heritability estimates for turkey lines selected for their corticosterone response to cold stress (high vs. low response), were estimated to be between 0.14 ± 0.09 and 0.25 ± 0.48^31^. In general, stress-induced plasma corticosterone levels align better with FCORT compared to basal plasma corticosterone levels^21,22^. This may explain why these reported heritabilities aligned closely with the heritability estimate for FCORT in the present study (0.21 ± 0.07). This moderate heritability estimate is consistent with expectations based on our previous finding that FCORT concentrations were different between purebred turkey lines housed under identical conditions, indicating some contribution of genetics to this trait^29^. FCORT is believed to represent the average circulating level of corticosterone in the plasma over the period of feather growth, which could explain the similarity in heritability estimates between other studies for plasma corticosterone and FCORT in the present study^31–34^. In general, it is found that stress-induced plasma corticosterone levels align better with FCORT compared to basal plasma corticosterone levels^21,22^. Since the mentioned heritability estimates for plasma corticosterone were based on stress-induced levels (restraint or temperature), this may explain why they aligned closely with the heritability estimate for FCORT in the present study.

Although there are no published heritability estimates for fault bars, it has been suggested that the propensity for developing feather fault bars has a genetic component. A cross-fostering experiment of barn swallows revealed that the likelihood of nestlings developing fault bars was not influenced by the experimental treatments (vitamin E supplementation and brood size manipulation), however, there was a significant influence of the nest of origin^28^. Since these nestlings were cross-fostered and not raised by their biological parents, it provides preliminary evidence that there is a heritable genetic component to fault bar presence. This hypothesis is supported by our findings as the heritability estimates for the fault bar traits were greater than zero, although the standard errors associated with these heritability estimates were high. To be more confident in the heritability estimates for these traits it would be beneficial to replicate the analysis with a larger sample size.

Before applying selection for novel traits in breeding programs, it is crucial to describe possible correlated responses^37,42^. Since fault bars are believed to be caused by acute perturbations (resulting in stimulation of the HPA axis), we would expect that the incidence and severity of these bars would be positively phenotypically and genetically correlated to the FCORT concentration in the feather. Other studies of different bird species have demonstrated that FCORT is elevated in feathers that contain fault bars compared to those that do not (e.g., in bald eagles^26^) and that exposure to stressors increases the number of fault bars (e.g., in broiler chickens^27^). Phenotypically, the strongest correlation was found between FCORT and fault bar index. This negative correlation suggests that higher FCORT is associated with fewer or less severe fault bars, contrary to expectations, although this correlation was still weak (-0.16). As discussed by Ganz et al.^43^ the keratin structure of the feather is what houses the corticosterone and so feathers with more keratin loss (from more severe fault bars) may actually have less measurable FCORT simply due to the keratin loss/breakage. However, other studies have found no phenotypic correlations between FCORT and fault bars^43,44^. Potentially, differences in how fault bars traits are assessed, scored and integrated (i.e., weighting of more severe scores) could have played a role.

Moderate genetic correlations were found between FCORT and total breast weight, breast meat yield, and fillet weight (−0.33 to −0.45). This relationship may be due to the high selection pressure to increase meat yield in domestic turkeys and the integral role that the HPA axis plays in growth and metabolism. Modern, more productive, poultry lines tend to have lower HPA axis activity compared to traditional lines or wild relatives^13^. This suggests that traditional lines are more robust or resilient than genetically selected lines, meaning that production potential is not/minimally compromised by perturbations (e.g., environmental changes, disease challenge)^2,3^. Faster growing lines of turkeys have been observed to be more sensitive to perturbations compared to smaller, slower-growing lines in response to e.g., transport stress^45^ or an E. coli challenge as indicated by greater body weight losses or mortality^46^. The trade-off between productivity and robustness is explained by resource allocation theory^4^. In brief, the energetic resources of an individual are limited, and selection for increased production allocates more resources for production traits (e.g., muscle growth), which leaves fewer resources for functional traits related to robustness or resilience^47^. Moreover, the primary role of glucocorticoids like corticosterone is to mobilize energy through protein catabolism^38^. Since high levels of protein catabolism would be detrimental to muscle growth and meat yield, selection for production will favor protein anabolism^48^. Therefore, it is logical that higher meat yields would be unfavorably correlated with FCORT. This relationship was also found in several experimental studies where elevated corticosterone levels result in decreased meat yield of poultry^49,50^ and in theunfavourable phenotypic correlations observed between FCORT and breast yield traits (−0.23 to −0.17) in the present study. Finally, there were significant positive partial phenotypic correlations between FCORT and drum weight and walking. This indicates that birds with higher FCORT have better walking ability and leg conformation, perhaps due to more proportioned/heavier drums, compared to birds with low FCORT. This may relate to selection for rapid growth and heavier breast weights resulting in structural problems in the legs and poor posture not suited for walking or standing^12^. However, these findings were not replicated in the genetic correlations and there is a need for further research with a larger sample size to test this hypothesis further.

There was also a positive genetic correlation between FCORT and ultimate pH. Ultimate pH is influenced by the amount of muscle glycogen which is broken down into lactic acid post-mortem and decreases the muscle pH^51,52^. Glucocorticoids, like corticosterone, can mobilize energy by glycogenolysis i.e., breaking down muscle glycogen into glucose^20^. With high glucocorticoid levels and thus more glycogenolysis, there would be lower levels of glycogen available in the muscle before slaughter to be broken down into lactic acid. Consequently, meat pH would not decrease as much^51^ explaining the positive correlation between FCORT and ultimate pH. This relationship could be a desirable consequence of selection for higher FCORT as it may help reduce the prevalence quality defects such as pale, soft, and exudative (PSE) meat^41,53–55^ which is associated with low meat pH. The correlation between FCORT and ultimate pH may also be influenced by the negative relationship between FCORT and breast yield where larger breast yield is associated with decreased pH and water-holding capacity.

Overall, this study presents initial heritability estimates and genetic and phenotypic correlation estimates for novel feather traits related to HPA axis activity. It should be reiterated that the sample size for this type of analysis was small (~ 1000 birds), and the results presented should be interpreted with caution. Moreover, the studied population included combined data from three purebred lines with line as a model effect to maximize the potential of our data and have more confidence in our estimates. When large enough sample sizes are available, future research should consider analyzing genetic line separately. In addition, structural equation models may be used to capture cause-and-effect mechanisms among the genetically correlated traits to improve our estimates and provide insights into how these estimates could be efficiently included in a turkey breeding program^37^.

To be included in a breeding program, high quality phenotypes are needed that accurately reflect the heritable trait and can be reliably and cost-effectively measured in large numbers of animals. The heritability estimates for the novel HPA axis traits presented in this study suggests it is possible to select for FCORT and/or fault bars, however, there are several challenges that need to be addressed before their inclusion in breeding programs under industrial conditions. Most importantly, further investigation is needed to determine what our ‘ideal’ phenotype is for these novel traits. Glucocorticoid levels can be detrimental when they are too low and when they are too high (Romero et al. 2009)^56^ and so FCORT is an intermediate trait but what this intermediate optimum would be is yet to be determined. Additionally, before novel traits can be included it is important to understand the correlations with already existing traits^37,57–59^. This study is, to the best of our knowledge, the first to investigate these relationships among FCORT, fault bar traits, and economically important traits in turkeys providing initial indications of a relationship between FCORT and production traits that may indicate robustness. However, it is important to further elucidate the relationship between the HPA axis and robustness by evaluating more robustness traits such as survival and confirmation as this is a complex concept and the number of traits in the current study is limited. Indeed, even glucocorticoid hormones like corticosterone are just one part of the HPA axis but there could be variation and opportunity for targeting other aspects of the system. For example, the responsiveness to adrenocorticotropic hormone (ACTH)^31^ or corticosterone carrying capacity that may vary between individuals and influence the biologically active “free” hormone fraction^60,61^.

From a practical point of view, measuring FCORT is time-consuming and relatively expensive and thus may not be feasible on a large scale. It might be more realistic from an industry perspective to use blood samples since these are collected routinely by breeding companies for genotyping, however, this measure is more sensitive to fluctuations and thus comes with its own challenges. Fault bars were included in this study as a non-invasive, inexpensive proxy that could be collected by human observers after initial training as similarly done for traits like walking score, breast conformation, or footpad dermatitis. Evaluating the presence versus absence of feather fault bars might be the most reliable and feasible of the fault bar traits since it does not require distinguishing between categories or spending time counting fault bars on each feather. These benefits would make FB fault bar presence the most logical proxy for FCORT but further validation, and potential alternative/automated methodologies for measurement, would be required. Although, based on the results of this study, the connection between FCORT and fault bars remains somewhat unclear with no genetic correlations between them which suggest fault bars would not be a good proxy. However, a more detailed understanding of fault bar development and different trait definitions would result in a different outcome.

Finally, once these above-mentioned issues are resolved, uptake of these traits in industrial breeding programs relies on clear communication and cost–benefit analysis ensuring a balanced breeding goal considering the relationships with existing traits. There needs to be a clear communication of the costs and potential benefits throughout the lifecycle of the bird. This is especially important because of some of the unfavourable correlations between FCORT/fault bars and highly economically important traits like breast meat yield. It will be necessary to demonstrate that selection for i.e., FCORT will not reduce gains in breast traits, or that the benefits from selecting for FCORT (i.e., improved survival, disease resistance, growth, etc.) will outweigh any reduction to genetic progress in yield. Nevertheless, the results from this study represent an essential first step to identifying novel traits related to HPA axis activity that could potentially be used in turkey breeding strategies to improve robustness.

## Methods

### Animals

Data were collected from male turkeys (N = 1131) from three genetic lines (A, B, and C) between May and November 2019. Turkeys were raised under identical housing and management conditions and processed at a commercial poultry processing facility in Ontario, Canada^62^. Line A was a dam-line selected for body weight and reproductive traits. Line B was a dam line selected for reproductive traits. Line C was a sire line selected for growth, yield, and feed efficiency. During processing, birds were electrically stunned and exsanguinated. Birds were then scalded, defeathered, and eviscerated before moving to a water chiller (2 h) and kept refrigerated for 24 h. After this, birds were deboned, and further meat quality measurements were taken. All protocols complied with the Canadian Council on Animal Care (CCAC) guidelines and were approved by the University of Guelph Animal Care Committee (AUP 3782). The study was conducted in accordance with relevant guidelines and regulations as well as the ARRIVE guidelines^63^.

### Feather sampling and measurements

At the processing plant, a feather (primary 9) was taken from each bird to measure FCORT after stunning and before scalding. Primary 9 was chosen for ease of identification and was previously validated to measure FCORT in domesticated turkeys using an ELISA kit^29^. A unique bird ID was recorded during feather collection to connect the feather with the other trait measurements. Feathers were rinsed briefly with ultrapure water to remove any debris that may obscure fault bars and/or influence FCORT, then allowed to dry overnight before being stored in paper envelopes until fault bar scoring and FCORT extraction.

Before being processed for FCORT quantification, fault bars were scored on each feather. First, it was recorded if fault bars were present on the feather (yes/no, FB presence). If fault bars were present, then fault bar incidence and severity were also recorded. Fault bar incidence was recorded as the number of fault bars present on each feather (count, FB incidence). Each feather was then scored on a 1–4 scale adapted from Sarasola and Jovani^24^ where 1 = no fault bars present, 2 = mild, 3 = moderate, 4 = severe (Supplementary Fig. S1). Mild fault bars are discernable by a visible discontinuity to the feather structure with no loss of keratin or translucency. Moderate fault bars are identified by a translucent band. Severe fault bars are identified by a translucent band accompanied by breakage of the barbules along the band. If multiple fault bar types were present on the same feather, then the highest score was recorded as fault bar severity (score 1–4, FB severity). Lastly, to capture both incidence and severity in one variable, a fault bar index was calculated as:

$$FB\;index = (X_{1} \times 0) + (X_{2} \times 1) + (X_{3} \times 2) + (X_{4} \times 3)$$

where *X*_1_, *X*_2_, *X*_3_ and *X*_4_ represent the number of fault bars recorded as score 1, 2, 3, 4, respectively, for each feather.

Feather samples were processed and analyzed using an ELISA (mouse anti-rabbit IgG, Corticosterone ELISA kit, number 501320, Cayman Chemicals, Cedarlane Labs, Canada) as described in Leishman et al.^29^. In brief, feather samples were minced with dissecting scissors and mechanically homogenized using a bead mill (Bead Blaster, Benchmark Scientific, Edison, NJ, USA). Feather powder was weighed using an analytical balance (15 ± 0.1 mg, model accu-124D Dual Range, Fisher Scientific, Toronto, Ontario, Canada). The powder was weighed into a test tube and 5 ml of methanol was added and sonicated in a water bath for 30 min. Samples were then moved to a shaking incubator at 50 °C for 12 h. After incubation, samples were vacuum filtered using Whatman #4 filter paper to separate the feather powder and methanol extract. During this process, the test tubes were rinsed with an additional 2 ml of methanol which was added to the extracted methanol (total = 7 ml). The methanol extract was evaporated using an evaporation plate under nitrogen gas at 40°. The extract residues were reconstituted in 0.5 ml of assay buffer immediately before being assayed. Samples were run in duplicate across 36 ELISA plates. The intra- and inter-assay coefficients of variation (CV) were 5.6% and 8.0%, respectively.

### Growth and efficiency traits

Body weight was measured at 12 weeks of age (BW12) and two days before slaughter (20–24 weeks of age, BW20). Walking score (WS20) was evaluated by trained observers at 20 weeks of age on a 1–6 scale where a higher score indicated better walking ability and leg conformation^64^. Feed conversion ratio (FCR) was calculated as total feed intake divided by weight gain^64^ using a real-time automated system for recording individual feed intake^65^.

### Carcass and meat quality traits

Carcass component weights were recorded using a scale at 24 h post-mortem. A proportion of carcasses were randomly selected and cut up into the major components as part of another project^41^. For these select carcasses, fillets (*Pectoralis major*), tenders (*Pectoralis minor*), thighs (bone-in, skin on), and drums (bone-in, skin on) were individually weighed, however a total breast meat weight (Breast_Total_) was estimated by adding the fillet and tender weight together. All remaining carcasses were processed separately, and only the Breast_Total_ was measured. Finally, breast meat yield (BMY, %) was calculated as Breast_Total_ divided BW20.

Meat quality measurements included pH, color, and physiochemical characteristics. At 24 h post-mortem, a pH measurement was taken from the cranial portion of the breast (pH_ultimate_ Portable pH meter, Hanna Instruments, Woonsocket, RI, USA). Trichromatic coordinates (L*, a*, and b*, CIE, 1976) were measured on the skinless breast fillet using a colorimeter with D50 illumination (Nix Pro Colorimeter, Hamilton, ON, CA).

At 24 h post-mortem (after weighing), two samples were taken from the breast fillet: a 13 ± 1 g sample to measure drip loss (DL) and a 200 g sample (approx. dimensions 7.5 × 7.5 × 4.5 cm) for measuring cooking loss (CL) and shear force (SF). Drip loss was calculated as the percent difference between the initial weight of the raw sample (13 ± 1 g) and final weight of the raw sample after refrigeration at 4 °C for 72 h. Cooking loss was calculated as the percent difference between the initial weight of the raw sample and final weight after cooking at 350 °C until an internal temperature of 72 °C was recorded using a thermocouple. The cooked samples were then refrigerated for 24 h. After allowing the samples to reach room temperature, SF was evaluated at six locations on the sample using a texture analyser (TA-XT2, Texture Technologies Corp., Hamilton, MA, USA) equipped with a Meullenet-Owens Razor Shear (MORS) blade^66^.

### Statistical analyses

Univariate and bivariate linear animal models were used to estimate variance components as follows:

$${\mathbf{y}} = {\mathbf{Xb}} + {\mathbf{Za}} + {\mathbf{e}}{,}$$

where **y** is the vector of observations sorted within animals; **b** is a vector including the fixed effects of hatch week-year (20 levels), age at slaughter (20–24 weeks), genetic line (3 levels: line A, B, and C), and initial sample weight (for DL and CL models only, g); **a** is a vector of additive genetic effects (breeding values), distributed as **a ~ *N*(0, A** $\otimes$** R)**, where **A** is the numerator relationship matrix including the inbreeding coefficients and **R** is the additive genetic variance–covariance matrix between the traits; **e** is the vector of residual effects which has a distribution of ${\mathbf{e}} \sim {\mathbf{N}}({\mathbf{0}},\;\sum\nolimits_{i}^{ + } {{\mathbf{E}}_{iy} } )$ E_iy_ indicates a *m*_*i*_x*m*_*i*_ matrix corresponding to the traits that were present for animal i, and m_i_ is the number of traits present for animal I; **X** and **Z** are incidence matrices for the fixed and random effects, respectively. The variance components were estimated using a restricted maximum likelihood method implemented in the BLUPF90 family of programs^67^.

The partial phenotypic correlation (r_P_) coefficients were calculated using SAS (version 9.4, SAS Institute Inc., Cary, NC, USA) to account for the fixed effects included in the linear animal model shown above. Significant partial phenotypic correlations were determined based on the *P* value (*P* < 0.05).

## Supplementary Information

Supplementary Information 1.

Supplementary Information 2.

## Data Availability

The datasets generated during and/or analysed during the current study are available from the corresponding author on reasonable request.
