# Supplementary material for: Genetic parameters of feather corticosterone and fault bars and correlations with production traits in turkeys (Meleagris gallopavo)
Source: Sci Rep. 2023 Jan 2;13:38. doi: 10.1038/s41598-022-26734-6 (PMC9807576; doi:10.1038/s41598-022-26734-6)
Supplement: Supplementary file 1 — Supplementary Information 1. [file 41598_2022_26734_MOESM1_ESM.docx]

**Supplementary Figure S1.** Feather fault bar scoring system (score 1-4) based on the fault bar strength gradient (light, medium and strong) adapted from Sarasola and Jovani (2006). Pictures by Anna R. Naim and Emily M. Leishman.

| **Score** | **Fault bar description** | **Visual description** |
| --- | --- | --- |
| 1 | No bar present. | 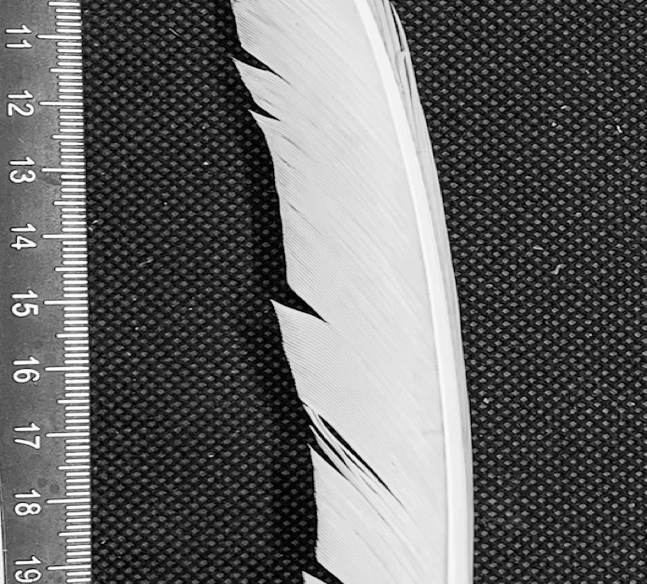 |
| 2 | Light bar present.  Narrow line across vanes that are greater than 5 mm in length.  Bar roughly perpendicular to the rachis. | 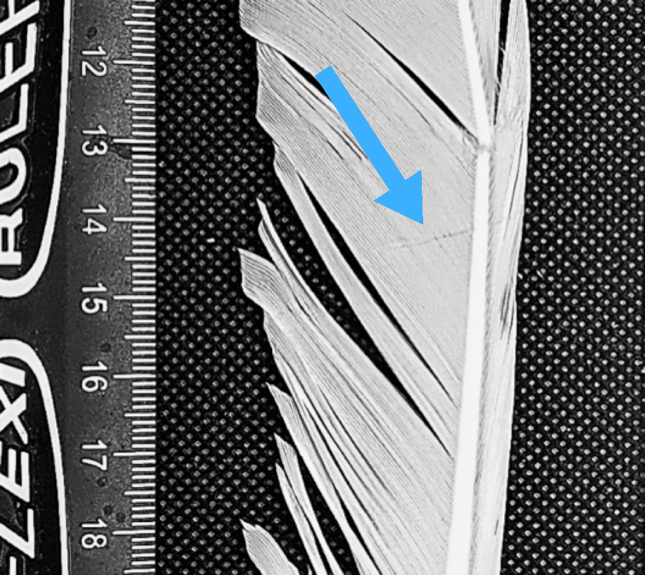 |
| 3 | Moderate bar present.  Thicker, translucent bar that is greater than 5 mm in length.  Line is translucent when feather is held up to a light source.  Bar roughly perpendicular to the rachis. | 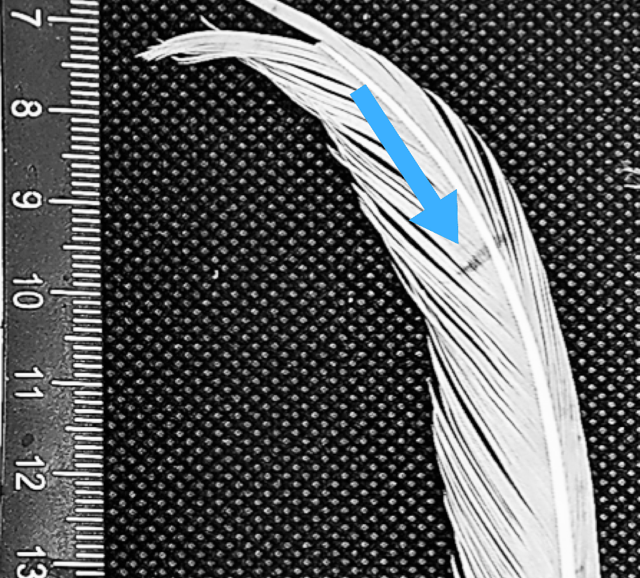 |
| 4 | Severe bar present.  Thicker bar (> 5 mm length) with barbule breakage along the bar line.  Line is typically translucent when feather is held up to a light source.  Bar roughly perpendicular to the rachis. | 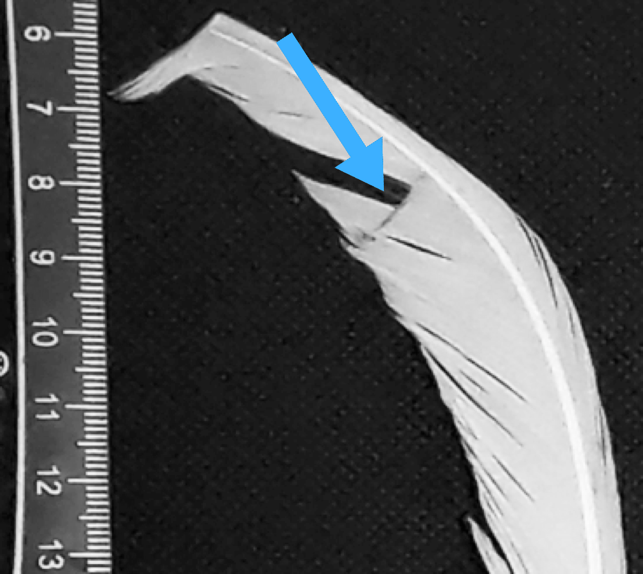 |
