# Supplementary material for: Genetic parameters of feather corticosterone and fault bars and correlations with production traits in turkeys (Meleagris gallopavo)
Source: Sci Rep. 2023 Jan 2;13:38. doi: 10.1038/s41598-022-26734-6 (PMC9807576; doi:10.1038/s41598-022-26734-6)
Supplement: Supplementary file 2 — Supplementary Information 2. [file 41598_2022_26734_MOESM2_ESM.docx]

**Supplementary Table S1.** Number of observations for the recorded traits separated by genetic line (A, B, and C).

|  | N_birds_ | | | |
| --- | --- | --- | --- | --- |
|  | All | Line A | Line B | Line C |
| FCORT | 1,131 | 429 | 346 | 356 |
| FB presence | 1,131 | 429 | 346 | 356 |
| FB incidence | 1,131 | 429 | 346 | 356 |
| FB severity | 1,131 | 429 | 346 | 356 |
| FB index | 1,131 | 429 | 346 | 356 |
| BW12 | 1,130 | 429 | 346 | 355 |
| BW20 | 1,114 | 426 | 337 | 351 |
| WS20 | 1,126 | 426 | 346 | 354 |
| FCR | 408 | 79 | 62 | 267 |
| Breast_Total_ | 1,060 | 424 | 321 | 315 |
| BMY | 1,045 | 423 | 312 | 310 |
| Fillets | 773 | 194 | 266 | 313 |
| Tenders | 771 | 193 | 264 | 314 |
| Thighs | 832 | 196 | 285 | 351 |
| Drums | 811 | 186 | 281 | 344 |
| L* | 1,068 | 401 | 320 | 347 |
| a* | 1,068 | 401 | 320 | 347 |
| b* | 1,068 | 401 | 320 | 347 |
| pH_ultimate_ | 834 | 195 | 286 | 353 |
| DL | 837 | 196 | 287 | 354 |
| CL | 834 | 196 | 288 | 350 |
| SF | 734 | 172 | 238 | 324 |
